# A Computerized System for Identifying Preschool Children with Suspected Neurodevelopmental Disorders in Early Childhood Education

**DOI:** 10.1192/j.eurpsy.2025.659

**Published:** 2025-08-26

**Authors:** G. O. E. Muitana, L. S. Silva, V. F. Martins, C. A. D. L. H. Amato

**Affiliations:** 1 Universidade Mackenzie, São Paulo, Brazil

## Abstract

**Introduction:**

Early childhood is a critical phase where the foundations for many essential skills are established. It is also a crucial time to monitor children’s development for early signs of neurodevelopmental disorders (NDD).

**Objectives:**

This study aimed to develop a computerized system to help preschool teachers identify risk signs of NDD.

**Methods:**

Phase 1: Development and validation of content and checklists - In the first stage, checklists were created to address four NDDs: ADHD, ID, ASD, and SLD. These were based on literature reviews and validated by four experts in neurodevelopmental disorders. The experts evaluated the checklists on two criteria: comprehensiveness and adequacy. Further assessment considered comprehension, objectivity, and precision of the checklist items, ensuring clarity for teachers using the system.

Phase 2: Development of the computerized system - The system was designed to assist early childhood educators in identifying children at risk for NDDs and supporting referral decisions, without diagnosing the disorders. Teachers begin by registering themselves and the child under observation. They then complete a checklist, indicating whether the child displays certain characteristics and the frequency of occurrence. A score of 1 indicates the presence of signs for ADHD, ID, ASD, or SLD, while 0 indicates absence. At the end of the process, the system generates a report with a risk level, which can be saved, edited, or printed for discussion with parents or specialists.

**Results:**

Phase 1 - Content validation showed high scores, particularly for ASD and ADHD (1.00), while SLD scored lower (0.75). The overall Content Validity Index (CVI) for all disorders was 0.91. Experts suggested minor adjustments to the ADHD and SLD sections, especially concerning developmental characteristics that may vary by age. The checklists were further evaluated for reliability, yielding an overall CVI of 0.87.

Phase 2 - The computerized system was built using a RESTful API in Node.js, with the Nest.js framework. The frontend was developed as a Single Page Application (SPA) using Angular, and PostgreSQL was used for data storage. The system includes data validation through the Zod library and user authentication via JWT. It is designed to be a precise, low-cost screening tool for identifying, not diagnosing, NDDs. The system does not differentiate between severity levels or subtypes of disorders, reinforcing its role as a first-line identification tool.

**Conclusions:**

Initial usability tests confirmed that the system is intuitive and suitable for its intended purpose. However, it is crucial to emphasize that the tool is not intended to operate autonomously. It supports, but does not replace, comprehensive clinical evaluations and the expertise of qualified professionals in diagnosing NDDs.

**Disclosure of Interest:**

None Declared

